# IPD-MHC: nomenclature requirements for the non-human major histocompatibility complex in the next-generation sequencing era

**DOI:** 10.1007/s00251-018-1072-4

**Published:** 2018-07-19

**Authors:** Giuseppe Maccari, James Robinson, Ronald E. Bontrop, Nel Otting, Natasja G. de Groot, Chak-Sum Ho, Keith T. Ballingall, Steven G. E. Marsh, John A. Hammond

**Affiliations:** 1 The Pirbright Institute, Woking, Surrey UK; 2 Anthony Nolan Research Institute, London, UK; 3 UCL Cancer Institute, Royal Free Campus, London, UK; 4 Biomedical Primate Research Centre, Rijswijk, The Netherlands; 5 Gift of Life Michigan, Ann Arbor, MI USA; 6 Moredun Research Institute, Midlothian, UK

**Keywords:** MHC, IPD-MHC, Database, Nomenclature, Comparative MHC, Variation

## Abstract

The IPD-MHC Database is the official repository for non-human MHC sequences, overseen and supported by the Comparative MHC Nomenclature Committee, providing access to curated MHC data and associated analysis tools. To address the increasing amount and complexity of data being submitted, an entirely upgraded version of the IPD-MHC Database was recently released to maintain IPD-MHC as the central platform for the comparison of curated MHC data. As a consequence, a new level of nomenclature standardisation is required between the different species to enable data submission and to allow the unambiguous inter- and intra-species comparison of alleles. However, any changes must retain the flexibility demanded by the unique biology of different taxonomic groups. Here, we describe the rationale for a standardised nomenclature system and summarise the changes that have been driven by the requirements of implementing the IPD-MHC database. This modified nomenclature system is essential to maintain the current functionality of IPD-MHC and provide a scalable future-proof database organisation to fully exploit the bioinformatic tools used for analysis.

## Introduction

It was evident, from early on, that within the MHC research community, nomenclature systems were required to prevent confusion when faced with a vast array of allele sequences and to avoid potential redundancy. In 1990, guidelines were proposed to provide a set of rules for the unambiguous naming of MHC alleles (Klein et al. 1990), and shortly afterwards, the Comparative MHC Nomenclature Committee was established, with the support of the International Society for Animal Genetics (ISAG) and the Veterinary Immunology Committee (VIC) of the International Union of Immunological Societies (IUIS). In order to promote the work of the MHC committee and provide a centralised repository of manually curated and annotated sequences, the Immuno Polymorphism Database (IPD) project was established in 2003 by the HLA Informatics Group of the Anthony Nolan Research Institute (Robinson et al. 2003, 2005). The IPD project (hosted by the European Bioinformatic Institute) is a set of related databases and associated analysis tools regarding the study of polymorphic genes that function within the immune system. Above all, the IPD-MHC Database stores MHC data that has been manually curated by experts according to the official nomenclature guidelines for each species. As such, IPD-MHC section curators and the Nomenclature Committee have a symbiotic relationship, each contributing to the development and improvement of MHC nomenclature.

During the last decade, the IPD-MHC Database has faced the challenge of constant growth in size, both in sequence numbers and taxonomic groups, and the associated demand for data access and unified bioinformatic tools. As new species were added, the problems of MHC nomenclature being specific to the needs of each section became more and more prevalent. Moreover, with the advent of high-throughput sequencing methods and the increasing number of sequences deposited, the work of curators - often relying on local analysis tools - became unsustainable. The lack of a unified organisation hampered the development of unified tools for data curation and comparison; cross-group comparison tools and a generic submission tool were impossible. Ultimately, this was creating and untenable demand from each section on the database developers, severely limiting the utility of IPD-MHC.

Other problems were more historical and reflected the complexity of particular sections. Several taxonomic groups (bovins, ovins, equids, swine, and canines) conserve non-standardised names either for genes or alleles. For example, in the canine group, the DLA abbreviation (Dog Leukocyte Antigen—recalling the human naming system) is based on the common species name and is used to name alleles shared between various organisms, included domestic dog, wolf, coyote and fox. This causes the allele nomenclature to be ambiguous, as a DLA gene or allele name may refer to a specific organism, a subgroup or all the organisms within that taxonomic group. This can be avoided by providing a rigorous subdivision by species, with a one-to-one relation between species and allele. However, where available, the database retains all the information related to breeds or hybridising species. For example, all organisms previously classified as *Canis lupus familiaris* are now listed within the *Canis lupus* species, featuring in the breed field. For an exhaustive review of the current MHC nomenclature for each section, please refer to the accompanying manuscript in this issue (Ballingall et al. 2018).

To address all these issues, a complete redesign and reorganisation of the data was undertaken. In 2016, IPD-MHC Database version 2.0 was released (Maccari et al. 2017). IPD-MHC 2.0 standardised the database organisation and provided a highly scalable framework that allowed the development of bespoke analysis tools. For example, IPD-MHC 2.0 now includes genomic sequences and allows the inter- and intra-species comparison of genomic and non-genomic allele sequences for the first time. This enhanced functionality has required a new level of standardisation in the MHC nomenclature between species and groups. These are changes that have been driven by the requirements of IPD-MHC and developed in concert with the MHC Nomenclature Committee. In this manuscript, the requirements for naming new alleles will be reviewed, and an overview of required changes in the taxonomic organisation of the MHC groups is presented, together with a set of core concepts to allow a more future-proof organisation of the IPD-MHC Database. The aim is to provide a universal bioinformatic framework, that is still flexible enough to deal with the complexities of MHC diversity in all jawed vertebrate species.

## MHC nomenclature requirements for IPD-MHC

**Fig. 1:**
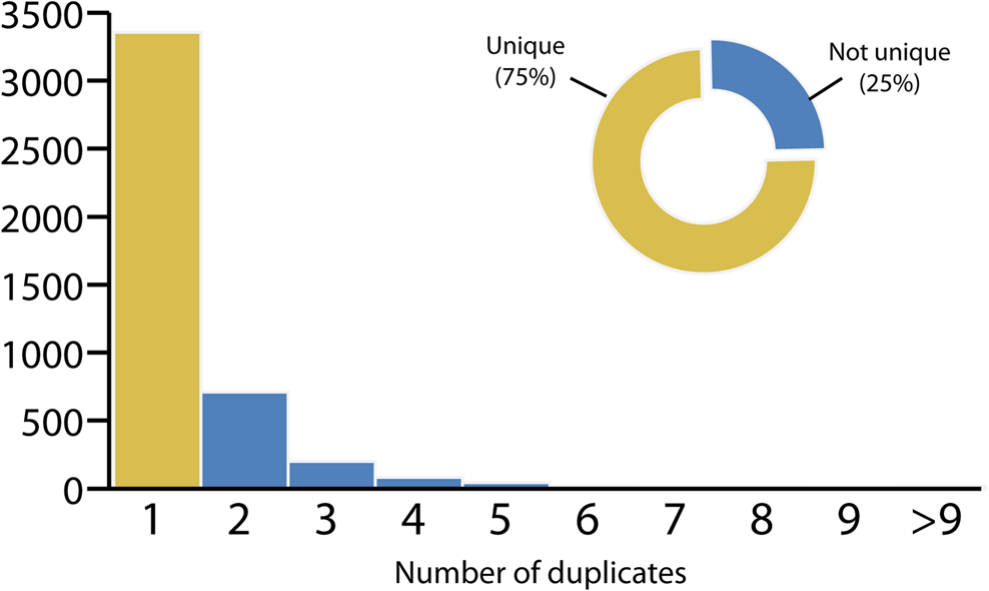
Four-letter codes distribution. Distribution of duplicate four-letter codes generated over all species belonging to the *Mammalia* class. Yellow, unique names; blue, duplicates

The realisation of the benefits that a standardised naming scheme for MHC alleles brings is not new. The MHC Nomenclature Committee proposed a set of naming rules with the aim of unambiguously identifying MHC genes and alleles (Klein et al. 1990; Ellis et al. 2006). In particular, it was suggested to prefix a four-letter code to the gene name, coined by abbreviating the species scientific name, maintaining a register of unique names to avoid ambiguities in the nomenclature. The relevance of a centralised nomenclature repository becomes evident when the combination of possible four-letter codes is analysed. Figure 1 shows the distribution of the number of duplicated four-letter codes within the *Mammalia* class, where about one quarter of the generated names present at least one repetition. However, while the four-letter code was adopted by most of the current MHC taxonomic groups, primarily by the non-human primate group, some of the suggested changes were not universally accepted, and researchers have for the most part retained the original names (Ellis et al. 2006), such as the *Canids*, *Felids* and *Suids* groups.

The MHC Nomenclature Committee is promoting the extended use of the four-letter code as an essential tool for the unambiguous comparison of data between species, together with a set of shared guidelines. These guidelines now represent the minimum requirements for the curation and naming of MHC alleles on IPD-MHC.

**Fig. 2:**
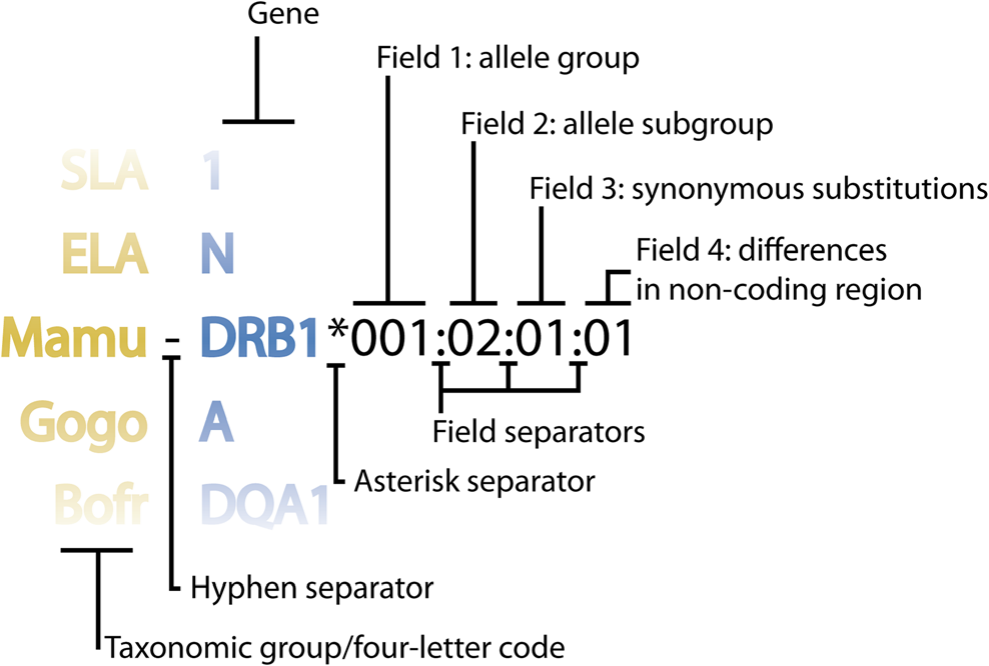
Non-human MHC nomenclature. Schematics of the nomenclature system for non-human MHC alleles. Yellow, the code representing the taxonomic group/species; blue, the gene. A hyphen divides the species four letter designation from the gene name, designated by capital letters and Arabic numerals. The gene designation is followed by an asterisk, and then by an allelic string composed of up to four sets of digits, separated by colons

- The IPD-MHC Database acts as the register of MHC species designations. When a request for the addition of an organism is received, a four-letter code is automatically generated from its scientific name by using the first two characters of the genus and species, respectively. If the name is already assigned, the next two characters of the species are used, until a unique name is generated.
- The species four-letter code will be used to unambiguously identify genes and alleles belonging to a specific group. When that group hosts one single species, the four-letter code can be considered superfluous and can be omitted. Furthermore, as the four-letter code is unique among all groups within the IPD-MHC Database, the group prefix may be dropped. For example, swine alleles belonging to locus 1 will be named SLA-1, instead of Susc-1 or SLA-susc-1; the water buffalo (*Bubalus bubalis*) will use the prefix Bubu-1, as the *Bovids* group enumerates more than one species.
- A hyphen divides the four-letter designation from the gene name, designated by capital letters and Arabic numerals. MHC class II genes are generally designated by the letter D, while MHC class I genes may be designated by any other letter or combination of letters. A second letter identifies families within MHC class II genes, while the third letter (A or B) identifies the chain that the sequence encodes. If an orthologous relationship to the HLA designation is obvious the same name should be used. The numbering of genes within each family (e.g., DRB1, DRB2, DRB3, etc.) should be sequential in the order of their initial discovery.
- The gene designation is followed by an asterisk as a separator and then by an allelic string. In order to facilitate inter species comparison of genomic data, guidelines from HLA nomenclature are adopted (Marsh et al. 2010). Allele names have a unique identifier formed by four sets of digits, separated by colons which allows an unambiguous identification of each of the four sets (Fig. 2). The first two sets are mandatory and describe the allelic group and the subgroup, respectively. Alleles whose numbers differ at this level exhibit one or more differences at the amino acid level. The third set of digits describes synonymous nucleotide substitutions within the coding regions. The last set of digits was recently added to support genomic data and describes sequence polymorphism within introns or untranslated regions. The number of digits for each set might vary from group to group, as the colons allow an unambiguous identification of the four sets.

## Changes in the taxonomic organisation

In order to align the IPD-MHC Database to the requirements listed above, a number of changes were made in the organism name assignment, some of these influencing the current nomenclature. The alleles belonging to *Ovins* taxonomic group are now represented with the four-letter code ‘Ovar’ to distinguish between the species belonging to that group: *Ovis aries* and *Ovis canadensis*. In the *Bovins* taxonomic group, *Bos taurus* ad *Bos indicus* were previously represented as separated species, each with its own unique four-letter code. In this version of the database, *B. taurus* and *B. indicus* share the name BoLA because many of the animals designated *B. indicus* are hybrids. This simplifies the search for alleles and also enables comparative analysis with complete datasets. Changes in the current taxonomic organisation are summarised in Table 1, together with a comparison of the previous system. It should be noted that for historical reasons, the human and Suids groups do not adopt the four-letter code designation. However, as each group includes only one species, there is no ambiguity in the allele nomenclature at the present time.

**Table 1:** Changes in the taxonomic organisation

Group	Scientific name	Common name	Previous designation	Current designation	Notes
OLA	*Ovis aries*	Domestic sheep	OLA	Ovar	
	*Ovis canadensis*	Bighorn sheep	OLA	Ovca	
BoLA	*Bos taurus*	Cattle	Bota	–	
	*Bos indicus*	Zebu	Boin	–	
	*Bos sp.*	Cattle	–	BoLA	*Bos sp.* substitutes and represents *Bos taurus*, *Bos indicus* and their hybrids
DLA	*Canis lupus*	Wolf	DLA	Calu	
	*Canis lupus familiaris*	Domestic dog	DLA	–	*Canis lupus familiaris* is merged with the *Canis lupus* species.
	*Canis rufus*	Red wolf	DLA	Caru	
	*Canis simensis*	Ethiopian wolf	DLA	Casi	
	*Canis latrans*	Coyote	DLA	Cala	
	*Canis lupus baileyi*	Mexican grey wolf	DLA	Caba	

The *Bovins* group highlights a problem common to most of the domesticated species, where the threshold between species and breed is not always evident and finding a common rule of thumb is not a trivial task. A striking example of the subtle difference between species and breed is the Canine taxonomic group, where different species have been enumerated under the single DLA name. Hybridization between *Canine* species has long been recognised both in the wild and in captivity, causing problems in the taxonomy, as hybrids are not normally thought of as species. As an example, a recent study (vonHoldt et al. 2011) indicates that the red wolf (*Canis rufus*) may be a hybrid species between grey wolves and coyotes, as it shares genomic sequences with both. Subsequent studies found contrasting evidence, leaving the debate still open. To alleviate the taxonomic ambiguity in the Canine group, a different four-letter code will be used to distinguish different species, in contrast with the previously adopted strategy. However, all canine alleles will still retain the current nomenclature, where the DLA prefix is used for all the species belonging to the group and alleles are named in a single series for each locus. Table 2 shows the status of each taxonomic group regarding the nomenclature guidelines listed above. For an exhaustive explanation of the state of the art of each taxonomic group, please refer to the accompanying review (Ballingall et al. 2018).

**Table 2:** List of taxonomic groups adopting the nomenclature requirements

Alias	Full name	Nomenclature standards		
		Four-letter code	Gene designation	Colons
HLA	Human	-^**1**^	Yes	Yes
NHP	Non-human primates	Yes	Yes	Yes
DLA	Canids	Yes	–	–
FISH	Salmonids	Yes	Yes	–
OLA	Ovids	Yes	Yes	Yes
BoLA	Bovins	Yes	Yes	Yes
ELA	Equids	Yes	Yes	Yes
SLA	Suids	-^**1**^	Yes	Yes
RT1	Murids	Yes	–	–
CHICKEN	Gallus	Yes	Yes	Yes

^1^Groups not conforming to the nomenclature requirements for historical reasons

## Summary and future development

The IPD-MHC team with the support of the Comparative MHC Nomenclature Committee have attempted to provide a common framework and set of guidelines to allow the hosting and analysis of MHC data from any species. The use of a common set of nomenclature rules across a range of species from divergent taxonomic groups, allows the storage and analysis of data in a biologically meaningful way. This allows the sharing of analysis tools and pipelines between taxonomic groups, thus improving the power and quality of data analysis. These standards on a common framework also provide future scalability of the database and readily facilitate the addition of new species and taxonomic groups as required by the research community. Furthermore, these changes represent the starting point for the development of a set of new tools for the analysis of genomic and non-genomic data, focussed on cross-species comparison. In conclusion, the suggested changes to the current nomenclature permit a much more future-proof organisation of IPD-MHC.

In 10 years, more than 10,000 sequences have been submitted to the IPD-MHC Database for curation and assignment of an official name prior to publication, providing a centralised repository for the official nomenclature and helping to drive database expansion in response to the most prominent topics in MHC research. As the amount of data increases and new species are included, inevitably, the nomenclature framework will have to adapt to meet the needs of the community. To this end, the IPD-MHC database represents the official resource for the scientific community, disseminating information and decisions made by the MHC Nomenclature Committee.
